# Improving access to assessments of early motor development in local languages: polish adaptation of the Early Motor Questionnaire

**DOI:** 10.1007/s00431-023-04895-4

**Published:** 2023-03-06

**Authors:** Zuzanna Laudańska, Magdalena Szmytke, Alicja Radkowska, Anna Malinowska-Korczak, Karolina Babis, David López Pérez, Przemysław Tomalski

**Affiliations:** 1 Neurocognitive Development Lab, Institute of Psychology, Polish Academy of Sciences, Warsaw, Poland; 2 Neurocognitive Development Lab, Faculty of Psychology, University of Warsaw, Warsaw, Poland

**Keywords:** Motor development, Infants, Parent reports, Pediatric physiotherapy, Early motor questionnaire

## Abstract

A child’s motor development progresses very dynamically. It is crucial to develop freely available parent-report measures of motor development that can be easily used globally to measure motor skills and identify children in need of interventions. This paper presents the adaptation and validation of the Early Motor Questionnaire, which consists of gross motor (GM), fine motor (FM), and perception–action integration (PA) subscales, to the Polish language (EMQ-PL). Study 1 (online, cross-sectional, *N* = 640) assessed psychometric properties of the EMQ-PL and its value in identifying children referred to physiotherapy. Results reveal excellent psychometric properties of the EMQ-PL and differences in GM and total age-independent scores between children that were and were not referred for physiotherapy. Study 2 (in-person assessment, longitudinal, *N* = 100) showed high correlations of GM and total scores with Alberta Infant Motor Scale.

*Conclusion*: Overall, the EMQ can be easily adapted to local languages and has the potential for use as a screening tool in global health contexts.

**Table:** 

**What is Known:** *• Parent-report questionnaires - especially those available free of charge - can potentially improve the rapid assessment of motor skills in young children worldwide.* *• Translation, adaptation and validation of freely available parent-report measures of motor development to local languages are important for local populations.*	
**What is New:** *• Early Motor Questionnaire can be easily adapted to local languages and has the potential for use as a screening tool in global health contexts.* *• The polish version of the Early Motor Questionnaire has excellent psychometric properties and highly correlates with infants’ age and Alberta Infant Motor Scale scores.*

**What is Known:**

*• Parent-report questionnaires - especially those available free of charge - can potentially improve the rapid assessment of motor skills in young children worldwide.*

*• Translation, adaptation and validation of freely available parent-report measures of motor development to local languages are important for local populations.*

**What is New:**

*• Early Motor Questionnaire can be easily adapted to local languages and has the potential for use as a screening tool in global health contexts.*

*• The polish version of the Early Motor Questionnaire has excellent psychometric properties and highly correlates with infants’ age and Alberta Infant Motor Scale scores.*

## Introduction

Motor development is a very dynamic process in the first few years of a child’s life. Acquisition of new motor skills changes the way infants can interact with their environment, and it is related to changes in other domains such as social interactions or language development (e.g., [1–9]). Although the cultural differences in child-rearing practices may influence the sequence of acquiring motor skills, the age of acquisition, and the developmental outcomes [10, 11], the traditional ages-and-stages approach to studying motor development is still widely applicable and important for the early detection of developmental problems. For example, the World Health Organization multi-center research showed that about 90% of children aged 4 to 24 months from Ghana, India, Norway, Oman, and the USA followed a common sequence of gross motor milestone acquisition [12]. Similarly, American Centers for Disease Control and Prevention has recently conducted a program to align empirically informed milestones to parent-completed surveillance tools and found that most typically developing children would achieve the developmental skills represented within a certain age window [13].

Reliable measures of motor development are essential for both clinical and research purposes. The most popular ways of assessing infants’ motor skills are the examiner-administered developmental scales such as the Bayley Scales of Infant Development [14], the Mullen Scales of Early Learning [15], the Peabody Developmental Gross Motor Scale [16, 17], and the Alberta Infant Motor Scales [18]. They are administered by a professional in a standardized procedure that usually requires dedicated space and a set of objects that are used in a series of tasks. These scales are well-documented and validated and appropriate for use in the general population. However, they are time-consuming and require expert training, and the scores rely on the infant’s performance in a given moment, which may not represent their full repertoire of skills. Furthermore, these tools are license based, thus, potential use involves a high initial cost of purchase of test materials as well as staff training and ongoing cost of scoring sheets [19]. For this reason, the possibility of using such tools more globally and in outreach populations (in languages other than English) is limited, especially considering the requirement of applying for the agreement of the publishing company to use their licensed materials.

Another approach to assessing motor milestones is parent-report questionnaires, which offer time-saving and economical alternatives, especially for screening purposes (for a review see [20]. These tools consist of a list of items that describe infants’ skills in a given area (e.g., reaching for different objects and proficiency in standing or crawling) (see example in Ages and Stages Questionnaire [21, 22]. The scores obtained by an infant are later compared to age-appropriate norms. Previous studies showed that parent report measures are accurate and convergent with experimenter-administered tools [21, 23, 24]. In practice, parent report measures are often used as an initial screening tool that is then followed by a direct observation measure if indicated by the parent report. Such a two-stage approach is both cost and time efficient. Therefore, availability of a reliable parent report measure is highly desirable even if decisions regarding developmental delays or treatments are ultimately based on more reliable observation measures. Age-appropriate checklists may be useful for parents to better understand the typical trajectories of motor development. In case of atypical performance of their child, they may seek professional consultants from early on [13]. This aspect seems to be of utmost importance nowadays since a recent report showed that infants born during the Covid-19 pandemic have lower gross and fine motor scores at the age of 6 months compared to a historical cohort of infants born before the pandemic [25]. Parent report questionnaires, especially those available free of charge, have the potential to improve rapid assessment of motor skills in youngest children worldwide. In comparison to experimenter-administered scales, the translation and adaptation of freely available questionnaires are much simpler and, for this reason, can enable cross-country comparisons as well as improve early screening of motor problems.

The Early Motor Questionnaire [26, 27] is a parent report measure of motor development for infants and toddlers aged 2–24 months based on observations of everyday situations. It consists of three subscales, gross motor (GM), fine motor (FM), and perception–action integration (PA) skills, and it takes approximately 15–20 min to fill in. It has been translated to other languages (Chinese, Italian, Swedish, and German; see https://www.onlinebabylab.com/emq for access to all versions), but the psychometric properties of the EMQ are unknown beyond the English language version. The original version shows robust concurrent and predictive validity with MSEL and PDSM and good test–retest reliability.

The EMQ, in contrast to other tools measuring motor development, is distributed freely on an open license basis. This aspect makes it an especially useful measure that can be adapted for a wide use in low- to middle-income countries. In addition, the EMQ has 128 items across three subscales (see Methods section for an overview of scoring) that capture various aspects of behaviors related to motor development; many of them are applicable from early on in infancy. This addresses a problem observed in other measurement tools such as MSEL, low number of items dedicated to the youngest infants, resulting in lower validity in the first half of the first year of life.

The current study aimed to adapt the original EMQ to Polish language (EMQ-PL) and to investigate its psychometric properties in a large sample (*N* = 640) of convenience collected online (study 1). The other parent report tool that is widely used in English-speaking populations, Ages and Stages Questionnaire [21, 22], is currently not available in Polish language. For this reason, the adaptation of a freely available parent report is important for the local population. Furthermore, our detailed description of translation and validation could become a guide for repeating this process for other languages. Moreover, we have applied age-independent scores [27] to investigate the effects of two variables that may affect motor development but have not been previously explored using the EMQ: sex differences and participation in pediatric physiotherapy (study 1). Sex differences have been previously reported at the level of motor activity with male infants being more active than females (Campbell and Eaton 1999) and with female infants having higher scores for fine motor skills compared to males [28]. However, the reports from non-English-speaking samples are scarce. Furthermore, we compared the scores of infants who were and were not referred for pediatric physiotherapy due to muscular hypertonia, hypotonia, or postural asymmetry. We have not evaluated the EMQ-PL as a tool to assess infants’ progress across participation in physiotherapy. Instead, we considered that in the case of those infants and toddlers that took part in physiotherapy, some aspects of motor development may arouse caregivers’ concern, thus, the EMQ could serve as a potential screening tool.

Finally, we also aimed to examine the concurrent validity of the EMQ-PL in comparison to the experimenter-administered Alberta Infant Motor Scale (AIMS) in another sample of typically developing infants during a longitudinal study consisting of 4 laboratory visits at the ages that reflect significant changes in gross motor development: when infants were around 4, 6, 9, and 12 months of age (study 2).

## Study 1

### Methods

#### Participants

A sample of convenience was recruited via social media (Facebook and Instagram) in Polish language. Overall, there were 759 complete responses; out of them, 640 met the following inclusion criteria: child’s age between 1.9 and 24.9 months, delivered at term (gestational age > or = 37 weeks), no older sibling with the diagnosis of autism spectrum disorder, and birth weight higher than 2500 g but lower than 4500 g. Younger siblings of children with autism spectrum disorder are considered at elevated likelihood of developmental disorders; thus, we decided to exclude them from the analysis. Similarly, as both low (< 2500 g) and high (> 4500 g) birth weight may contribute to later adverse outcomes [29], we included only infants with birth weight within the normal range (see Table 1). Approximately 30% of caregivers declared that their infants were referred for pediatric physiotherapy (specifically, our questions were as follows: Has your child attended sessions with a physiotherapist/rehabilitation specialist due to motor development difficulties? If so, for what reason, for how long (e.g., months) and with what frequency*?*). The most common reasons were hypertonia, hypotonia, and postural asymmetry. The online questionnaire was filled in mostly by mothers (637 mothers and 3 fathers). The majority of the caregivers had completed higher education: 17 had a PhD (2.65%), 558 held a university degree (87.19%), 64 completed high school (10%) and 1 completed vocational school (0.16%). This distribution is above national levels of education in Poland, as according to Eurostat [30] over 40% of Poles aged 25–34 years have tertiary educational attainment, whereas around 6% have less than primary, primary, or lower secondary level of education. Caregivers’ subjective assessment of their family’s socioeconomic situation indicated that the majority of our sample belonged to the middle class: 144 (22.5%) described their economic situation as “very good,” 344 (53.8%) as “good,” 137 (21.4%) as “ok,” 11 (1.7%) as “not good,” and 2 (0.3%) as “very bad” (2 respondents, 0.3%, declined providing answer to this question).

**Table 1:** Sample characteristics in study 1

**Age in months**	***N*** **(% group)**	**Mean age in months (SD)**	**Min. age**	**Max. age**	**Females (% group)**	**Participation in physiotherapy (% group)**
**Entire sample**	640 (100%)	12.73 (5.83)	1.92	24.90	298 (46.6%)	202 (31.6%)
**1.9–6.0**	92 (14.38%)	4.40 (1.14)	1.92	5.97	36 (39.1%)	30 (32.6%)
**6.01–12.0**	217 (33.91%)	8.99 (1.76)	6.03	11.98	105 (48.4%)	69 (31.8%)
**12.01–18.0**	195 (30.47%)	15.03 (1.75)	12.01	18.00	93 (47.7%)	62 (31.8%)
**18.01–25.0**	136 (21.25%)	21.05 (1.94)	18.07	24.90	64 (47.1%)	41 (30.1%)

#### Questionnaire translation and adaptation

Permission to translate and validate the EMQ was obtained from the author of the original English version. The EMQ was translated into Polish independently by 5 authors of this paper (ZL, MS, AR, AMK, and PT). All discrepancies were discussed and resolved through group consensus. Untranslatable expressions were replaced by Polish equivalents, which sometimes required providing illustrative examples. The consensus translation was reviewed by a specialist in Polish linguistics for clarity and jargon-free wording. Several mothers of young children were interviewed to assess the understanding of the instructions and all statements. The feedback was used to construct the final version of the EMQ. A professional translator conducted a back-translation (Polish to English). A comparison of the back-translation and original versions of the questionnaire was performed, and the results were used to develop the final consensus version. The EMQ-PL and back-translation were approved by the first author of the original version [26].

The EMQ-PL follows the English original in the order of items. It is a list of questions that asks the parent about the presence or absence of a given infant behavior at the time of assessment. It uses a 5-point scale ranging from − 2 to + 2 to quantify parents’ certainty. A behavior is rated − 2 if the parent is sure the child does not show the behavior yet, − 1 if the child probably does not show the behavior yet, 0 if the parent is unsure whether the child shows the behavior or not, + 1 when the child probably shows the behavior, and + 2 if the parent remembers a particular instance where the child exhibited the behavior (see Table 2). This is a retrospective tool: parents are asked to assess whether their child exhibited given behavior at any time in the past. Following the original version, the EMQ-PL scores are computed separately for the gross motor (GM), fine motor (FM), and perception–action integration (PA) domains and for the total score. There are 49 items on the GM scale (possible score range: from − 98 to + 98), 48 on the FM scale (possible score range: from − 94 to + 94), and 31 on the PA scale (possible score range: from − 62 to + 62, possible total score range: from − 254 to + 254).

**Table 2:** Instructions for parents in the EMQ and EMQ-PL

**Score**	**− 2**	**− 1**	**0**	**1**	**2**
**English version**	Sure that child does not show behavior	Child probably does not show behavior yet	Unsure whether child could do this or not	Child probably shows this behavior	Sure that child shows this behavior and remember a particular instance
**Polish version**	Z pewnością dziecko NIE przejawiało takiego zachowania	Dziecko prawdopodobnie NIE przejawiało takiego zachowania	Brak pewności, czy dziecko przejawia takie zachowanie	Dziecko prawdopodobnie przejawiało takie zachowanie	Z pewnością dziecko przejawiało takie zachowanie i pamiętasz konkretną sytuację

#### Data collection

The EMQ-PL questionnaire and a demographics and health-related survey were completed anonymously by caregivers of infants and toddlers aged 1.9–25 months using Qualtrics software (Qualtrics, Provo, UT, USA). Due to Covid-19 restrictions and national lockdown, the data collection was conducted entirely online. The caregivers were also asked to report whether their child was referred to a pediatric physiotherapist. If yes, they were asked to provide the reason for it. On average, the caregivers were filling in the questionnaire (together with additional demographic questions) in 45.2 min. However, standard deviations were large (SD = 202.8 min and max value: 70.27 h), which is probably the result of answering the questions not on one sitting but across several days. The adaptation was carried out as part of a larger research project, which obtained clearance from the ethics committee at the Institute of Psychology, Polish Academy of Sciences.

#### Data analysis

To investigate the psychometric properties of the EMQ-PL in the group of typically developing infants, we calculated Cronbach’s alpha. Pearson’s correlation coefficients were used to check the relations between the EMQ-PL subscale scores and the participant age. Partial correlations controlling for infant’s age were used to check the relations between subscales and the total score of the EMQ-PL. To verify whether the EMQ-PL has equally good psychometric properties across all tested ages, we calculated Cronbach’s alpha separately for four age groups representing every half-year (infants aged 1.9–6.0, 6.01–12.0, 12.01–18.0, and 18.01–24.9 months). Data analysis was conducted in IBM SPSS Statistics 28; data was visualized using R [31], RStudio [32], and ggplot2 package [33]).

**Fig. 1:**
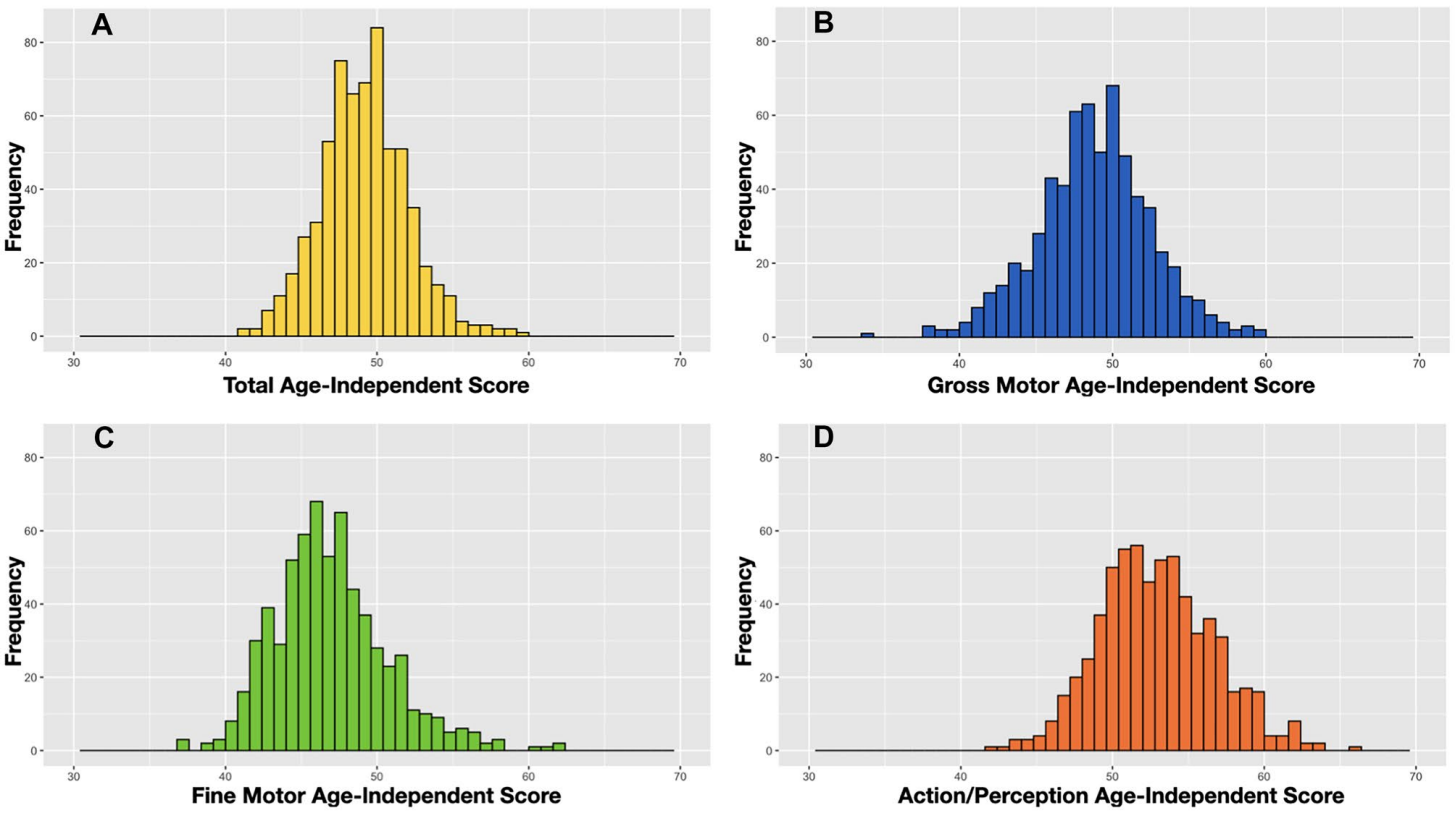
Histograms showing distributions of age-independent scores: **A** total, **B** gross motor (GM), **C** fine motor (FM), and **D** action/perception (PA)

To investigate differences in EMQ-PL scores between infants who were and were not referred for physiotherapy as well as potential sex differences across the wide age range, we have calculated age-independent scores (see [27] for detailed description and equations and Fig. 1 for the distribution of scores in our sample). Next, we used independent *t*-tests, which are robust to large discrepancies in group size, to check the effects of sex and referral for physiotherapy on EMQ-PL age-independent scores.

### Results

#### Descriptive statistics

Descriptive statistics for EMQ-PL raw scores are presented in Table 3. Several participants in the two oldest groups reached maximum score in the GM subscale (first half of the second year: *N* = 1, second half of the second year: *N* = 3). Similarly, some participants from the oldest group reached max score for PA subscale (*N* = 9). In contrast, none of the infants reached max score in the FM subscale: the highest score was 84, which is 10 points below the max score.

**Table 3:** Descriptive statistics of EMQ-PL scores

**Age in months**	***N***	**Total score**			**Gross motor**			**Fine motor**			**Perception/action**		
		**Mean (SD)**	**Min**	**Max**	**Mean (SD)**	**Min**	**Max**	**Mean (SD)**	**Min**	**Max**	**Mean (SD)**	**Min**	**Max**
**Entire sample**	640	31.16 (115.33)	−219	244	12.93 (52.28)	−86	98	0.15 (36.11)	−85	84	18.06 (29.65)	−54	62
**1.9–6.0**	92	−146.65 (29.78)	−219	− 73	−61.98 (10.48)	−86	−28	−57.65 (13.06)	−85	−31	−24.02 (9.36)	−54	−10
**6.01–12.0**	217	−37.94 (53.90)	−138	99	−18.64 (27.18)	−67	59	−18.05 (16.70)	−58	27	−1.24 (14.68)	−24	47
**12.01–18.0**	195	96.11 (51.48)	−39	212	41.63 (29.25)	−35	98	19.09 (15.87)	−12	65	35.39 (13.85)	−6	59
**18.01–25.0**	136	166.41 (29.64)	72	244	72.94 (14.88)	−8	98	41.11 (15.18)	−1	84	52.46 (6,98)	28	62

#### Internal consistency

The Polish EMQ had excellent internal consistency for all subscales in all age groups (see Table 4). Cronbach’s alphas in all age groups were above 0.9 for the total score and above 0.7 for the three subscales (Cronbach’s alpha score of 1.0 is the max possible value). The lowest consistency was observed for GM in the first half of the first year of life (0.781) and for PA in the oldest group aged 18 to 25 months (0.783), which are still very high scores.

**Table 4:** Cronbach’s alphas for EMQ-PL in each age group

**Age in months**	***N***	**Total score**	**GM**	**FM**	**PA**
**Entire sample**	640	0.988	0.977	0.959	0.961
**1.9–6.0**	92	0.928	0.781	0.840	0.826
**6.01–12.0**	217	0.962	0.934	0.873	0.886
**12.01–18.0**	195	0.953	0.937	0.848	0.865
**18.01–25.0**	136	0.909	0.856	0.849	0.783

#### Correlations with age

**Fig. 2:**
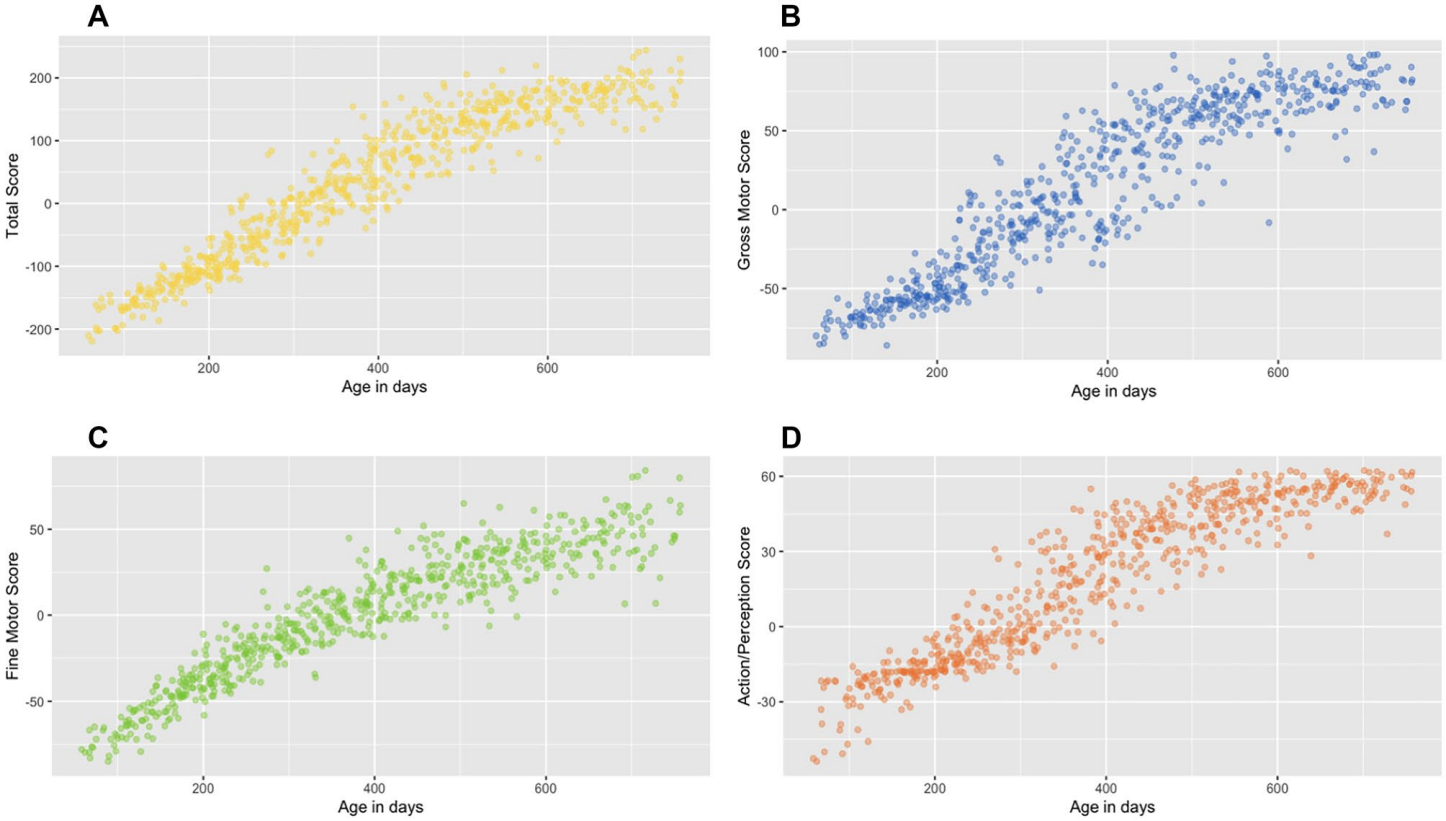
Correlations of the EMQ-PL total score (panel **A**) and subscales’ scores (**B**-GM, **C**-FM, and **D**-PA) with age

The age was positively correlated with all EMQ-PL raw scores (see Fig. 2): total score: *r*(638) = 0.946, *p* < 0.001; GM: *r*(638) = 0.921, *p* < 0.001; FM: *r*(638) = 0.925, *p* < 0.001; PA: *r*(638) = 0.928, *p* < 0.001.

#### Correlations between the subscales

We investigated if there were relations between the EMQ subscales. Partial correlations with age as a controlled variable were used to investigate the correlations between the subscales. All three subscales significantly correlated with each other and with the total score (Table 5). Interestingly, FM and PA were more correlated with each other than with GM score, which highlights the sensitivity of the EMQ to the relations between object manipulation and perceptual skills.

**Table 5:** Partial correlation coefficients between subscales and total score controlling for age

**Variable**		**Total**	**GM**	**FM**
**GM**	Pearson’s *r*	0.867	-	
	*p*-value	< 0.001	-	
**FM**	Pearson’s *r*	0.821	0.507	-
	*p*-value	< 0.001	< 0.001	-
**PA**	Pearson’s *r*	0.771	0.467	0.608
	*p*-value	< 0.001	< 0.001	< 0.001

#### Sex differences

To check if there were sex differences among the different subscales of the EMQ-PL, we used age-independent scores. The results did not show any sex differences in the total score, *t*(638) =  − 1.21 and *p* = 0.227, or any of the subscales: GM: *t*(638) =  − 1.19, *p* = 0.236; FM: *t*(638) =  − 0.26, *p* = 0.795; PA: *t*(638) =  − 1.27, *p* = 0.203.

Similarly, when we conducted separate analyses for four age groups representing every half-year, we have not observed sex differences in any of the EMQ-PL scores (see Table 6).

**Table 6:** Comparison between EMQ-PL scores across sexes in 4 age groups

**Age in months**	***N***	**Total score**	**GM**	**FM**	**PA**
**1.9–6.0**	92 (56 boys)	*t*(90) = −0.813, *p* = 0.419	*t*(90) = −0.982, *p* = 0.329	*t*(90) = −1.022, *p* = 0.310	*t*(90) = −0.063, *p* = 0.950
**6.01–12.0**	217 (112 boys)	*t*(215) = 0.465, *p* = 0.643	*t*(215) = 0.408, *p* = 0.684	*t*(215) = 0.671 *p* = 0.503	*t*(215) = 0.188, *p* = 0.851
**12.01–18.0**	195 (102 boys)	*t*(193) = −1.427, *p* = 0.155	*t*(193) = −1.459, *p* = 0.146	*t*(193) = −0.554, *p* = 0.580	*t*(193) = −1.587, *p* = 0.114
**18.01–25.0**	136 (72 boys)	*t*(134) = −0.634, *p* = 0.527	*t*(134) = −0.476, *p* = 0.635	*t*(134) = −0.541, *p* = 0.589	*t*(134) = −0.499, *p* = 0.618

#### Pediatric physiotherapy: age-independent scores

**Fig. 3:**
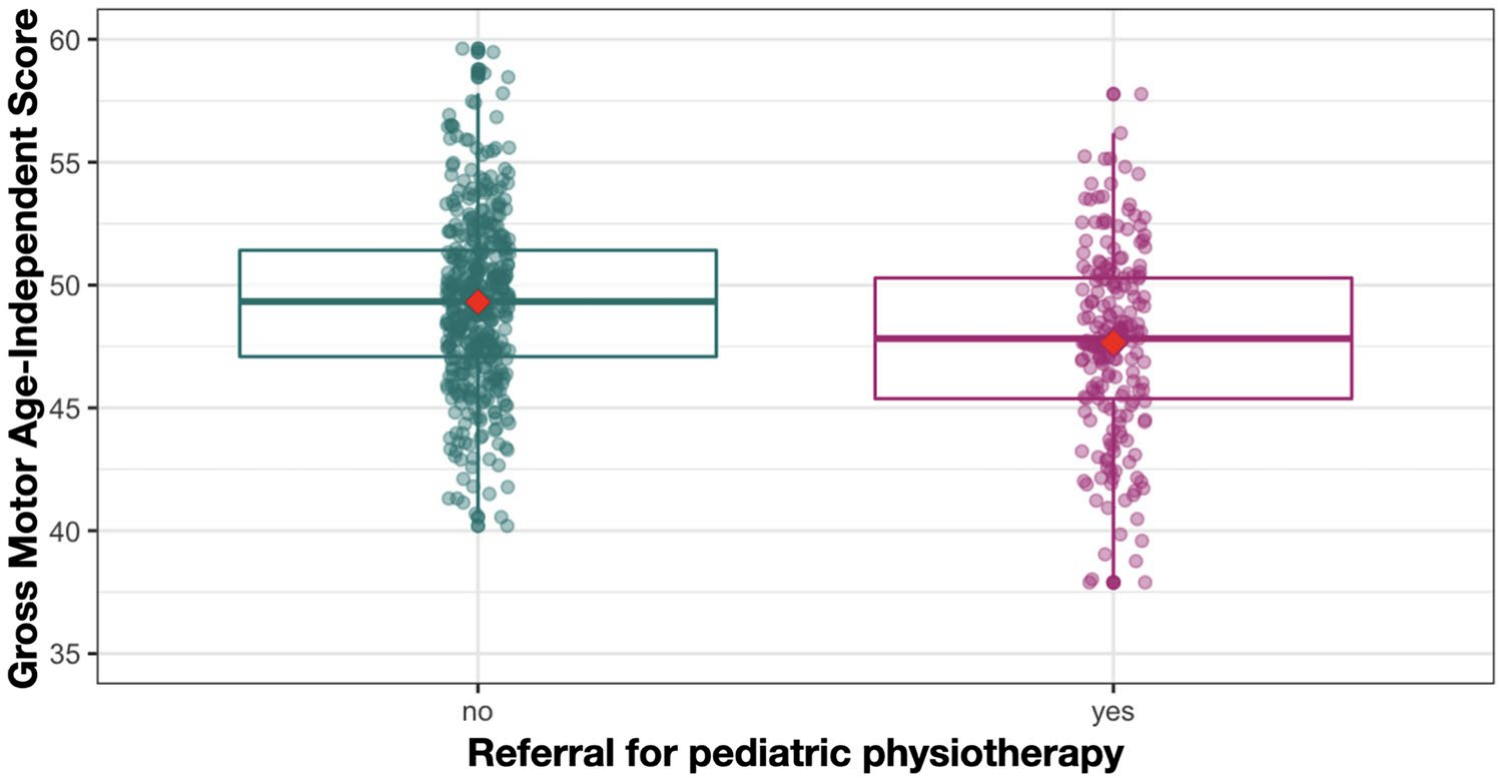
Boxplots representing age-independent gross motor scores in groups that were and were not referred for pediatric physiotherapy. Dots represent each infant’s GM scores, and red diamond indicates mean value

To assess whether the EMQ-PL may be suitable as a screening tool, we compared the age-independent scores of infants who were and were not referred for pediatric physiotherapy (see Table 7). Infants who were referred for pediatric physiotherapy had significantly lower GM scores (*N* = 202) compared to those who did not (*N* = 438), two-sided *t*-test *t*(638) = 5.63, *p* < 0.001, and Cohen’s *d* = 0.48 (see Fig. 3). Similarly, infants who were referred for physiotherapy had lower total score: *t*(638) = 3.44, *p* < 0.001, and Cohen’s *d* = 0.29. No differences were found for the other two subscales: FM: *t*(638) = 0.45, *p* = 0.652; PA: *t*(638) = 0.01, *p* = 0.989.

**Table 7:** Age-independent scores for infants referred and not referred for physiotherapy

**Age in months**	**Referred for physiotherapy**	**Not referred for physiotherapy**
**Total**	Range: 41.43–58.3, *M* = 48.59, *SD* = 2.91	Range: 41.36–59.36, *M* = 49.42, *SD* = 2.83
**GM**	Range: 33.71–57.77*, M* = 47.58, *SD* = 3.95	Range: 40.19–59.62,* M* = 49.32, *SD* = 3.46
**FM**	Range: 36.93– 61.95, *M* = 46.82, *SD* = 3.81	Range: 37.53–61.61, *M* = 46.97, *SD* = 3.71
**PA**	Range: 44.18–63.95, *M* = 52.92, *SD* = 3.68	Range: 42.33–65.75, *M* = 52,92, *SD* = 3.80

### Discussion

Here, we presented the process of adaptation of the Early Motor Questionnaire to Polish language (EMQ-PL) and its validation in a large sample (*N* = 640) of Polish infants aged 2 to 24 months. We showed that EMQ has excellent overall internal consistency and good reliability of its subscales across all age groups. The internal consistency in the youngest group of infants, especially the GM subscale, turned out to be smaller yet still acceptable. In addition, we have shown that raw scores on all subscales are highly correlated with infants’ age as well as with each other. Furthermore, using age-independent scores [27], we found that the EMQ-PL was sensitive at the group level to differentiate between infants who were and were not referred for physiotherapy. Furthermore, in our sample, we have not found any sex-related differences in any of the subscales. There are discrepancies in the literature reporting sex differences in early development [28, 34]. Our relatively big sample consisted of a similar proportion of males and females with similar socioeconomic status and did not reveal significant differences in any given scales. The results based on comparisons of four age groups indicate that the norms may be applied independently of sex.

One of the limitations related to this study is the fact that we cannot say with full confidence where participants were located at the moment of filling in the questionnaire, since such questions were not included in the survey. Our recruitment has focused on social media in Polish language, websites, fora, and parenting blogs, so it can be assumed that we reached out predominantly to parents residing in Poland.

In the online study, we were not able to investigate the concurrent validity of any of the EMQ-PL subscales. For this reason, we recruited an independent sample of Polish infants for a lab-based study and examined the concurrent validity of the EMQ-PL gross motor and total scores and the experimenter-administered Alberta Infant Motor Scale [18] at four time points across the first year of life when infants were around 4, 6, 9, and 12 months of age. We selected these ages as they reflect significant changes in motor control and gross motor development. The longitudinal design of study 2 will also enable us to examine the stability of the age-independent scores across time.

## Study 2

### Methods

#### Participants

An independent sample of parent–child dyads participated in the longitudinal project investigating the relations between motor and communicative development during early social interactions. Participants were invited to the lab when the infants were around 4 (T1), 6 (T2), 9 (T3), and 12 (T4) months of age (see Table 8 for age distribution). Overall, 104 families from the metropolitan area of Warsaw (Poland) took part in the study, and 100 dyads provided questionnaire data at minimum 1 time point. Caregivers’ subjective assessment of their family’s economic situation indicated that the majority of our sample belonged to the middle class: 38 (38%) described their economic situation as “very good,” 48 (48%) as “good,” 10 (10%) as “ok,” and 1 (1%) as “not good” (3, 3%, missing data). The majority of the caregivers had completed higher education: 3 (3%) had a PhD, 91 (91%) held a university degree, and 4 (4%) completed high school (2, 2%, missing data). The questionnaire in the lab was filled in mostly by mothers (T1: 4 fathers, T2: 6 fathers, T3: 6 fathers, and T4: 7 fathers). We have excluded from the analysis an infant that was born preterm and had low birth weight (*N* = 1) and an infant with too high birth weight and diagnosed with fetal macrosomia (*N* = 1). None of the infants that provided data had an older sibling with confirmed diagnosis of autism spectrum disorder. Overall, 98 infants across 321 visits were included in the final sample. Some participants provided incomplete data (see Table 8 for the number of data points available at each time point).

**Table 8:** Age distribution of the final sample in study 2

**Age in months**	***N***	**Mean age (SD)**	**Min age**	**Max age**
**T1**	74	4.34 (0.28)	3.90	4.90
**T2**	83	6.60 (0.41)	6.00	7.80
**T3**	80	9.07 (0.37)	8.20	10.20
**T4**	84	12.14 (0.52)	11.50	14.50

#### Alberta infant motor scale (AIMS)

The Alberta Infant Motor Scale [18] is an observational assessment tool for measuring motor development of infants aged 0 to 18 months. It consists of 4 subscales that list skills in four body positions: prone, supine, sit, and stand. For each of the four positions, an experimenter needs to identify and score the least and most mature items observed during the assessment. The items between the least and most mature of the observed items represent the infant’s possible motor repertoire in that position, also considered their “window” of current skills. Each item within this window is then scored by either “observed” or “not observed” by the experimenter. All of the “observed” scores on a given subscale are then summed to obtain a positional score. Finally, all positional scores are summed to obtain a total AIMS score.

#### Data collection

During the lab visits, the caregivers completed the paper version of the EMQ-PL, and the experimenters administered the AIMS. The examiners were blind to the EMQ-PL scores at the time of the AIMS assessment. The caregivers were completing the questionnaire within 10–15 min. These tasks were part of longer testing sessions which also included infant-parent interactions in wearable motion trackers and head cameras (data not presented here). For their participation, families received a diploma and a small gift (a baby book). The project obtained clearance from the ethics committee at the Institute of Psychology, Polish Academy of Sciences.

#### Data analysis

The relationships between the gross motor and total scores of the EMQ-PL and the total score of the AIMS were analyzed using Pearson’s correlation coefficients. Since the study had a longitudinal design and most infants provided data at more than 1 time point, the analyses regarding validity were conducted separately for each time point to avoid autocorrelations. Furthermore, to examine the stability of the EMQ-PL age-independent scores, we calculated Pearson’s correlation coefficients between time points for each subscale and for the total score. Data analysis was conducted in IBM SPSS Statistics 28, and data was visualized using R, RStudio, and ggplot2 package.

### Results

**Fig. 4:**
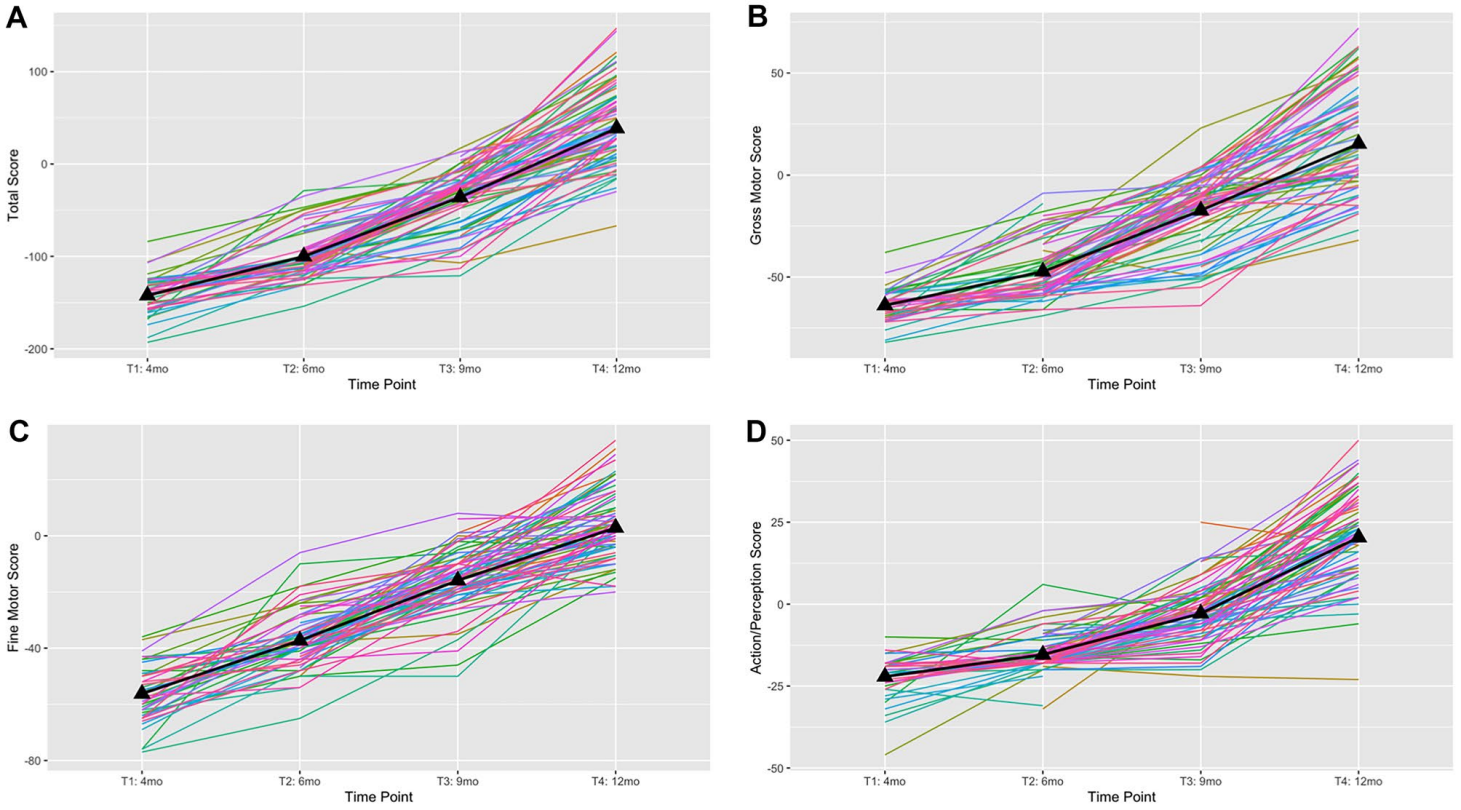
The developmental change in the EMQ-PL total score (panel **A**) and subscales’ scores (**B**-GM, **C**-FM, and **D**-PA) with age. Colorful lines indicate scores of individual infants, and black lines with triangles indicate mean values

#### Concurrent validity of the EMQ-PL gross motor and total scores and the AIMS score

We assessed the concurrent validity of the EMQ-PL gross motor and total raw scores and the AIMS total score (see all descriptive statistics in Table 9 and Fig. 4 for the developmental change in the EMQ-PL raw scores across time points) by checking Pearson’s correlations at each time point. Since the AIMS does not have subscales that correspond to fine motor or action/perception development, these two subscales of the EMQ-PL were not included in this analysis.

**Table 9:** EMQ-PL and AIMS descriptive statistics

**Age in months**	**EMQ total score**			**Gross,motor**			**Fine motor**			**Perception/action**			**AIMS total score**		
	**Mean (SD)**	**Min**	**Max**	**Mean (SD)**	**Min**	**Max**	**Mean (SD)**	**Min**	**Max**	**Mean (SD)**	**Min**	**Max**	**Mean (SD)**	**Min**	**Max**
**T1**	−142.25 (20.51)	−193	−84	−63.90 (8.28)	−82	−38	−56.23 (8.28)	−78	−36	−22.11 (6.83)	−46	−10	13.78 (3.69)	5	26
**T2**	−100.00 (23.59)	−154	−29	−37.285 (10.08)	−69	−9	−37.29 (10.09)	−65	−6	−15.39 (5.71)	−32	6	24.96 (7.80)	13	47
**T3**	−36.17 (31.52)	−121	17	−17.42 (18.18)	−64	23	−15.90 (10.93)	−50	8	−2.84 (8.59)	−22	25	40.19 (10.68)	21	56
**T4**	38.59 (43.27)	−67	147	15.34 (25.61)	−32	72	2.87 (12.47)	−27	34	20.38 (13.69)	−23	44	51.37 (4.99)	39	58

**Fig. 5:**
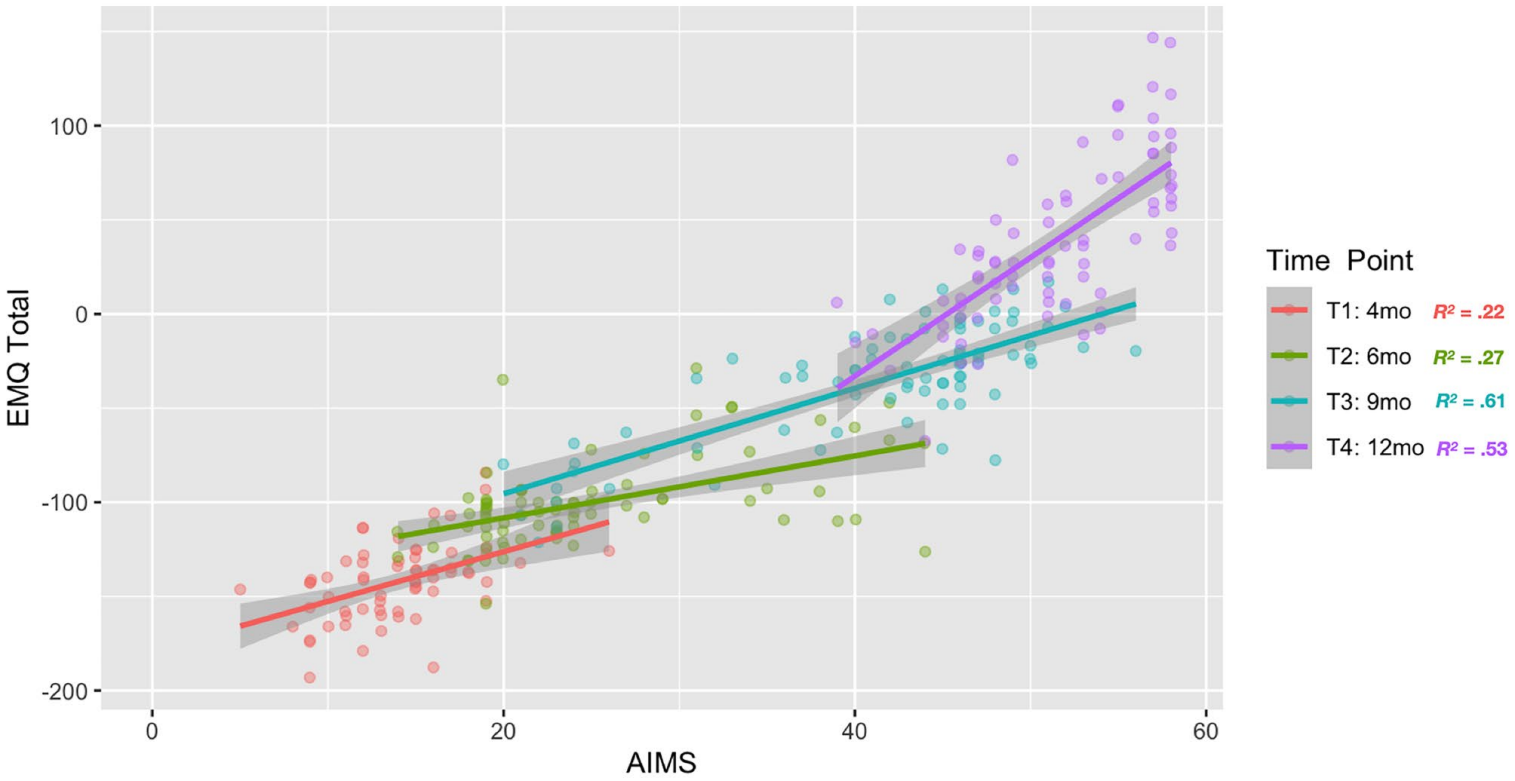
EMQ-PL total raw score by AIMS total score. Dots indicate scores of individual infants, and colors of dots indicate which time point data were collected

The EMQ-PL total scores were highly positively correlated with the AIMS total score at a corresponding time point (T1: *r*(73) = 0.474, *p* < 0.001; T2: *r*(84) = 0.524, *p* < 0.001; T3: *r*(82) = 0.781, *p* < 0.001; T4: *r*(83) = 0.729, *p* < 0.001; see Fig. 5).

**Fig. 6:**
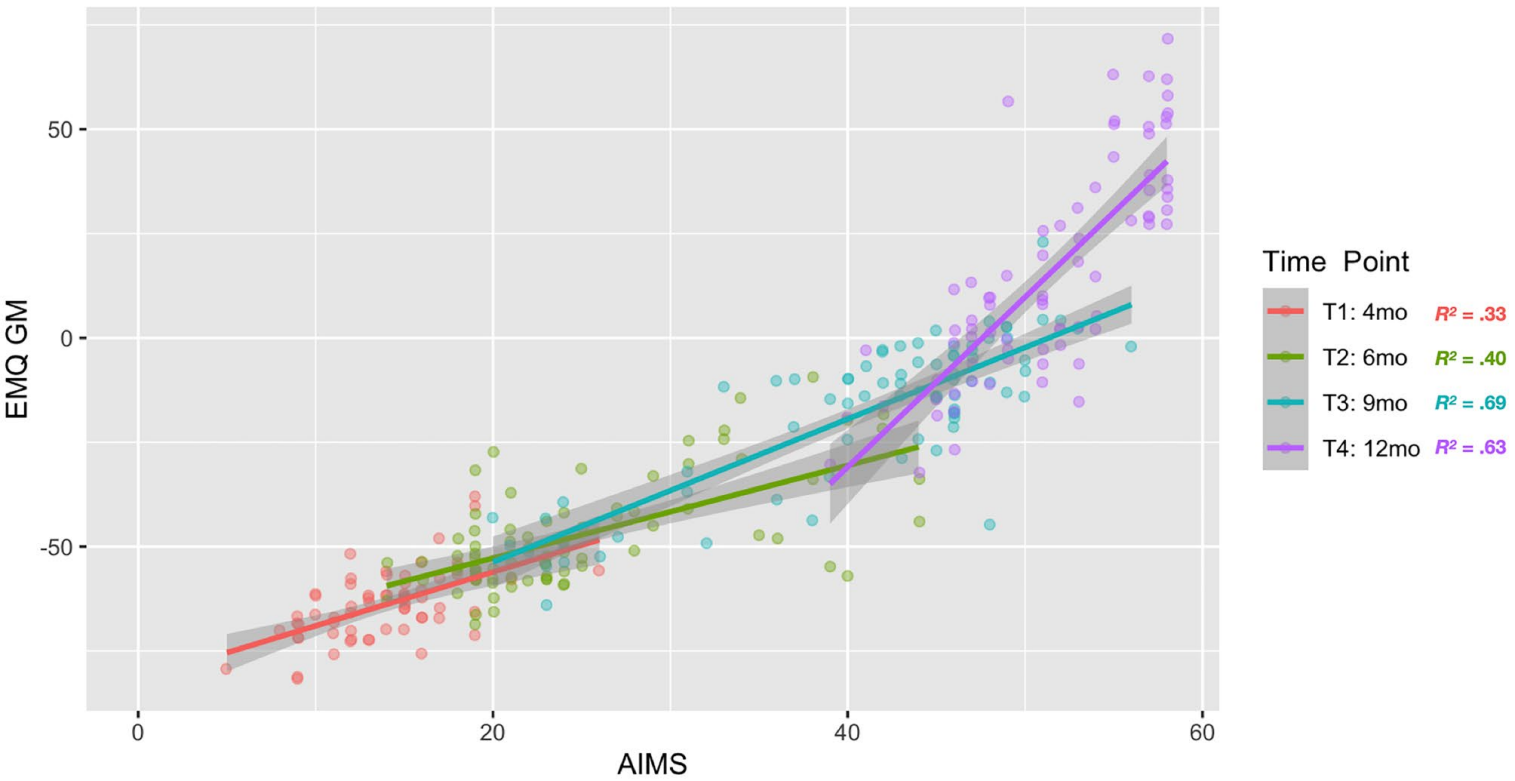
EMQ-PL gross motor raw score by AIMS total score. Dots indicate scores of individual infants, and colors of dots indicate which time point data were collected

Similarly, the EMQ-PL gross motor scores were highly positively correlated with the AIMS total score at a corresponding time point (T1: *r*(73) = 0.574, *p* < 0.001; T2: *r*(84) = 0.635, *p* < 0.001; T3: *r*(82) = 0.828, *p* < 0.001; T4: *r*(83) = 0.796, *p* < 0.001; see Fig. 6). The high correlations between EMQ-PL and AIMS scores confirm high concurrent validity of these two tools.

#### Stability of age-independent scores

To examine the stability of the EMQ-PL age-independent scores, we calculated correlations between time points for the GM (Table 10), FM (Table 11), and PA (Table 12) subscales as well as for the total score (Table 13). Our findings indicate that the age-independent scores show stability over time in case of total score and gross motor score. Also, to some extent, we observed the stability of the fine motor score; however, the correlations between scores obtained at 4 months and 12 months as well as 6 months and 12 months were not significant. In the case of action/perception subscale, correlations between the first time point and later ones were not significant. However, the age-independent scores of this subscale seem to be more stable across the second half of the first year of life (especially between 9 and 12 months).

**Table 10:** Correlation coefficients of EMQ-PL gross motor subscale between time points

	**GM T1**	**GM T2**	**GM T3**
**GM T2**	***r*(44) = 0.530, *p* < 0.001**	**-**	
**GM T3**	***r*(49) = 0.459, *p* < 0.001**	*r***(64) = 0.562, *p* < 0.001**	**-**
**GM T4**	***r*(48) = 0.544, *p* < 0.001**	***r*(64) = 0.436, *p* < 0.001**	*r***(69) = 0.769, *p* < 0.001**

Bold emphasis indicate significant correlations

**Table 11:** Correlation coefficients of EMQ-PL fine motor subscale between time points

	**FM T1**	**FM T2**	**FM T3**
**FM T2**	***r*(44) = 0.325,** ***p*** **= 0.031**	-	
**FM T3**	***r*(49) = 0.348,** ***p*** **= 0.014**	***r*(64) = 0.429,** ***p*** **< 0.001**	-
**FM T4**	*r*(48) = 0.213, *p* = 0.146	*r*(64) = 0.069, *p* = 0.585	***r*(69) = 0.471,** ***p*** **< 0.001**

Bold emphasis indicate significant correlations

**Table 12:** Correlation coefficients of EMQ-PL action/perception subscale between time points

	**PA T1**	**PA T2**	**PA T3**
**PA T2**	*r*(44) = 0.108, *p* = 0.484	-	
**PA T3**	*r*(49) = 0.224, *p* = 0.122	*r*(64) = 0.234, *p* = 0.063	-
**PA T4**	*r*(48) = 0.199, *p* = 0.174	***r*(64) = 0.254,** ***p*** **= 0.043**	***r*(69) = 0.486,** ***p*** **< 0.001**

Bold emphasis indicate significant correlations

**Table 13:** Correlation coefficients of EMQ-PL total score between time points

	**Total T1**	**Total T2**	**Total T3**
**Total T2**	***r*(44) = 0.473,** ***p *< 0.001**	-	
**Total T3**	***r*(49) = 0.467,** ***p* < 0.001**	***r*(64) = 0.516**, ***p*** **< 0.001**	-
**Total T4**	***r*(48) = 0.444,** ***p*** **= 0.002**	***r*(64) = 0.404,** ***p* < 0.001**	***r*(69) = 0.723,* p*** **< 0.001**

Bold emphasis indicate significant correlations

## General discussion

The Early Motor Questionnaire [26] is a parent report measure of infants and toddlers motor skills. It is based on everyday situations, can be completed in under 20 min, and is shown to have good validity compared to examiner-administered measures in the USA sample [26]. In the present study, we translated and adapted the Early Motor Questionnaire (EMQ) to the Polish language (EMQ-PL), confirmed its reliability, and investigated its concurrent validity with the Alberta Infant Motor Scale in a Polish sample. We also explored whether there are any sex differences in the EMQ-PL scores and whether this tool is sensitive to common early motor problems requiring participation in pediatric physiotherapy, such as higher or lower than typical motor tone or asymmetry.

We observed good reliability of the EMQ-PL in a relatively large sample of infants aged 1.9 to 25 months. The EMQ-PL had an excellent internal consistency also when breaking the sample down by age groups, which shows its robustness across the first 2 years of life. It is especially important with regard to the youngest infants (in the first half of the first year of life) as a common problem of many developmental scales and questionnaires [15, 35] is the low number of items dedicated to this group and as a result: low validity of measurement. Furthermore, the EMQ-PL was also highly correlated with infants’ age. To the best of our knowledge, this is the first study showing the psychometric characteristics of a non-English version of the EMQ. Moreover, our data showed strong correlations of the total score and all subscales’ scores with age, suggesting that standardized age norms could be established for the EMQ-PL. This is in line with the results from the USA sample [26].

We also investigated the concurrent validity of parent reports on early motor development in the EMQ-PL with the examiner-administered measure of gross motor development (AIMS). The total scores on both scales were positively correlated, which is in line with previous studies in English-speaking populations showing the usability of the parent-report measures (e.g., [24, 26, 36–42]). The gross motor score as well as the total scores was highly positively correlated with the AIMS score. However, it should be noted that both tools measure early motor development, and that each of them has a slightly different main focus. The AIMS is focused entirely on the gross motor development, and it investigates in detail postural development, tracking progress across subscales (prone, supine, sitting, and standing), whereas the EMQ includes a more broad set of questions related to gross, fine, and perceptual development, which limits the scope of direct comparisons. Nonetheless, our comparison between EMQ and AIMS adds to the previous comparisons of the EMQ to the Mullen Scales of Early Learning and the Peabody Developmental Gross Motor Scale, meaning the EMQ has now been compared to three out of four major direct assessment tools (future studies should also include comparisons between the EMQ and the Bayley Scales of Infant Development).

Furthermore, we observed differences in gross motor scores between infants who were and were not referred for pediatric physiotherapy. For this reason, the EMQ-PL may have a potential as a screening tool for identifying infants who are in need of early intervention. In our study, we asked the caregivers to provide information about infants’ referral for pediatric physiotherapy, as we considered it a marker of parental concern about infant motor development. We found out that the EMQ-PL was sensitive at the group level to differentiate between infants who were and were not referred for physiotherapy. However, that was true only for the gross motor score, which suggests that this subscale alone could work as a screening tool. Overall, this suggests that the EMQ-PL could be potentially used as an inexpensive, time-effective, and easy-to-use screening tool to identify children in need of further clinical assessment [20], which could be considered an equivalent to level 1 screening instruments that are used to identify children at risk in the general population, in contrast to level 2 screening instruments used to identify the risk level among children already considered to be at increased likelihood of developing certain problems (e.g., [43]). Future studies should investigate the sensitivity of the EMQ-PL to different types of early motor problems and in various clinical populations in a similar way as the AIMS [44] as well as its suitability for assessing the infant’s progress along the course of professional intervention. Furthermore, it would be beneficial to establish the screening confidence of the EMQ by comparing it to the Ages and Stages Questionnaires [22], which is a widely used parent report screening tool. Unfortunately, the ASQ in Polish language is currently not available for purchase, so we could not plan such comparisons in the present study.

In contrast to some other studies (e.g., [28, 34]), we have not observed any sex differences in motor scores in our sample. Several studies reported mixed results depending on the measured aspect of motor skills and/or children’s age. For example, Veldman and colleagues (2018) showed sex differences in locomotion but only in children < 20 months (Australian sample, tool: Peabody Developmental Gross Motor Scale II, *N* = 178, for an older group this effect was not observed). In the same sample, the authors did not find differences between girls and boys for a stationary subtest. Özal et al. [45] did not report any sex differences in a large sample of 2,042 Turkish children aged 0–72 months (tool: Denver II Developmental Test for Turkey). Dinkel and Snyder [28] reported the effects of sex for fine motor skills but not for gross motor skills (sample from the USA, *N* = 29, tool: Bayley Scales of Infant and Toddler Development III). Furthermore, sex differences in the age of motor milestones’ acquisition were not observed in the WHO multi-center research conducted in Ghana, India, Norway, Oman, and the USA (*N* = 1433, longitudinal assessment, de Onis [47], tool: own standardized assessment protocol as in [46]. The differences between the discussed studies could be related to the measurement tools, variables of interest (fine vs. gross motor skills), sample size, and diversity in terms of age and socioeconomic status. Nonetheless, the sex differences in motor development are not fully clear. However, our results using the EMQ-PL suggest that for this tool, the same norms could be used for females and males.

In line with Smith and Libertus [27] study, we showed stability of age-independent scores over time for total score, gross motor score, and, to some extent, for fine motor score, with infants who had high age-independent scores at the first time point also having high age-independent scores at other time points. The action/perception score showed the most overall variability but was stable in the second half of the first year of life.

Some limitations of the study should be noted. First, in the concurrent validity study, we have not included the entire range of ages that can be tested with the EMQ-PL; therefore, it is yet to be determined for infants older than 12 months of age. Second, since Alberta Infant Motor Scale can only be used to assess infants’ development in the gross motor skills, we have not been able to measure the concurrent validity of the EMQ-PL fine motor and action/perception scores. Third, in the recruitment, we have relied on a convenience sample of middle class families and caregivers interested in participating in academic research, so the results may not be representative of all infants (e.g., these caregivers may be more vigilant for delays in their child’s motor development and therefore seek professional help more often than a general population). Fourth, we consider our analysis regarding the differences between infants who were and were not referred for pediatric physiotherapy exploratory. Since in the online study we were not able to collect more detailed information regarding the reason of the physiotherapy referral (or even details regarding how necessary the initial referral was), the course and duration of the intervention, and the suggested changes regarding home exercises or infant handling practices, it is yet to be investigated how sensitive the EMQ-PL is to various types of problems in motor development, especially taking into consideration that although the effect size of the physiotherapy referral was moderate, the difference between the two groups was rather low. We believe that this could be related to high variability of our participants in both groups; also, in terms of the potential severity of motor problems, we likely captured a relatively large number of children who needed some minor adjustments in day-to-day handling practices as well as those in need of longer interventions. Furthermore, the caregivers whose infants attend physiotherapeutic consultations may better understand their infants’ motor repertoire, since specialists instructed them to observe particular aspects of motor development, such as particular body positions. This, in turn, could affect how they understand items in the EMQ-PL and how they fill in the answers.

## Summary

This study presented the adaptation of the Early Motor Questionnaire to the Polish language (EMQ-PL, available for download here: https://www.onlinebabylab.com/emq) and its validation in two samples. In the first study (online data collection), we observed excellent psychometric properties of the EMQ-PL and positive correlations with age in a sample of infants aged 2 to 24 months. We did not observe any sex differences on any subscale (using age-independent scores). Infants who were referred to a physiotherapist for a consultation showed significantly lower gross motor and total age-independent scores. In the second study (in-person), we found that the Alberta Infant Motor Scale total scores were highly correlated with the EMQ-PL total and gross motor scores in infants aged around 4, 6, 9, and 12 months. Overall, our studies show that the EMQ-PL is a good parent report tool to measure early motor development and potentially can be used as a screening tool for motor development for infants and toddlers.

## Data Availability

Dataset created for study 1 is available in OSF: https://osf.io/q3fwr/?view_only=ce7ad80d3ea64335a8ae1b9ecc38441f. The dataset presented in study 2 will be available upon request from the corresponding authors following an embargo period from the date of publication to allow for the finalization of the ongoing longitudinal project. Requests to access the dataset should be directed to ptomalski@psych.pan.pl.
